# A Review of Monitoring Methods for Cerebral Blood Oxygen Saturation

**DOI:** 10.3390/healthcare9091104

**Published:** 2021-08-26

**Authors:** Wentao Zhong, Zhong Ji, Changlong Sun

**Affiliations:** 1 College of Bioengineering, Chongqing University, Chongqing 400044, China; 20145946@cqu.edu.cn (W.Z.); 20145939@cqu.edu.cn (C.S.); 2 Key Laboratory of Biorheological Science and Technology, Chongqing University, Ministry of Education, Chongqing 400044, China

**Keywords:** cerebral blood oxygen saturation, monitoring, SjvO_2_, PbtO_2_, NIRS, non-invasive

## Abstract

In recent years, cerebral blood oxygen saturation has become a key indicator during the perioperative period. Cerebral blood oxygen saturation monitoring is conducive to the early diagnosis and treatment of cerebral ischemia and hypoxia. The present study discusses the three most extensively used clinical methods for cerebral blood oxygen saturation monitoring from different aspects: working principles, relevant parameters, current situations of research, commonly used equipment, and relative advantages of different methods. Furthermore, through comprehensive comparisons of the methods, we find that near-infrared spectroscopy (NIRS) technology has significant potentials and broad applications prospects in terms of cerebral oxygen saturation monitoring. Despite the current NIRS technology, the only bedside non-invasive cerebral oxygen saturation monitoring technology, still has many defects, it is more in line with the future development trend in the field of medical and health, and will become the main method gradually.

## 1. Introduction

The brain is one of the most essential organs in the human body and it is of great significance to the life activities of the human body. Although the weight of the brain only accounts for approximately 2% of the total body weight, cerebral blood flow (CBF) volume accounts for 15–20% of the cardiac outputs and roughly 20% of the whole-body oxygen consumption [1]. Moreover, the cerebral oxygen consumption of a six-year-old child accounts for approximately 50% of the whole-body oxygen consumption [2]. Recently, cerebral blood oxygen saturation has been increasingly recognized as an important physiological indicator, and been applied in surgical anesthesia, neonatal monitoring, and clinical treatment of patients with brain injury. An absence of cerebral blood oxygen saturation monitoring probably allow brain tissue and functions to become damaged and even lead to irreversible consequences. If the blood and oxygen supply to the brain is completely cut off, the individual will lose consciousness in 8–15 s; lasting 6–10 min, anoxic conditions will lead to the irreversible brain injury and brain death in severe cases [3]. Therefore, an effective method for cerebral blood oxygen saturation monitoring during surgery process and the postoperative period is needed to protect cerebral function, and improve postoperative cognitive function.

The commonly used cerebral blood oxygen saturation monitoring methods in hospitals include jugular venous oxygen saturation (SjvO_2_) monitoring, electroencephalogram (EEG), event-related potentials (ERPs), and transcranial doppler ultrasonography (TCD) [4,5,6]. Among these, SjvO_2_ is the earliest monitoring method used in the clinic; however, since most of these methods are invasive or indirect measurement techniques that cannot reflect the cerebral blood and oxygen supply in real-time, it is possible to get false-negative or false-positive results, which make interpretations and differentiations more difficult [7]. To eliminate these defects, researchers proposed new methods for cerebral blood oxygen saturation monitoring. Invasive methods include brain tissue partial pressure of oxygen (PbtO_2_) monitoring, and non-invasive methods include positron emission tomography (PET), functional magnetic resonance imaging (fMRI), and near-infrared spectroscopy (NIRS). Recent studies have reported that these new monitoring methods tend to be more effective and reliable than conventional ones and are more suitable for cerebral blood oxygen saturation monitoring and relevant research. Based on different principles and techniques, these new methods have their own advantages and defects in the following aspects: temporal resolution, spatial resolution, cost, detection scope, noise artifacts, and applicable environment [8,9,10].

The present study discusses the working principle, detection performance, and research progress of different cerebral blood oxygen saturation monitoring methods in surgical and postoperative settings.

## 2. Cerebral Blood Oxygen Saturation Monitoring Methods

One of the primary purposes of cerebral blood oxygen saturation monitoring is to avoid secondary brain injury caused by anoxia or ischemia. The earliest monitoring method for the damaged brain is intracranial pressure (ICP) and cerebral perfusion pressure (CPP) monitoring [11,12] but ICP and CPP monitoring are inadequate for the avoidance of secondary brain injury, because these methods cannot fully reflect CBF or oxygen saturation. Owing to the scientific and technical progress achieved in recent years, more measurement methods for brain tissue oxygenation have been described, some of which have been carried out under conditions not limited to pathological.

### 2.1. Jugular Venous Oxygen Saturation Monitoring

The jugular bulb oxygen saturation is designated by SjvO_2_. The jugular bulb is preferred for blood sampling to monitor SjvO_2_. The jugular bulb locating below the skull base is where the jugular vein is dilated. SjvO_2_, which can be used for the indirect evaluation of brain tissue oxygen consumption, reflects a dynamic balance between the whole-brain oxygen supply and consumption. Therefore, SjvO_2_ is considered as a useful indicator of the relationship between whole-brain blood flow and brain metabolism [13].

#### 2.1.1. Working Principles

**Figure 1:**
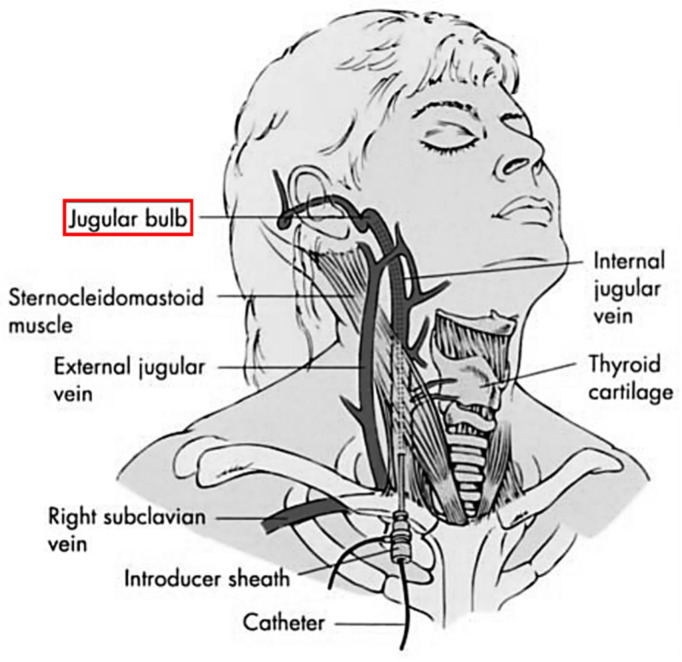
Schematic of the cerebral veins [13].

SjvO_2_ is the percentage of oxygenated hemoglobin, and is related to the whole-brain oxygen supply and consumption. SjvO_2_, in conjunction with other parameters, can offer an indirect evaluation of brain tissue oxygen metabolism. A fiber catheter is placed in the jugular bulb (Figure 1) to extract blood samples for indirect or continuous measurement of SjvO_2_ [14]. Based on these measurements, Fick’s formula is used to calculate the brain tissue oxygen metabolism for representing the whole-brain oxygen saturation. SjvO_2_ monitoring is a non-qualitative monitoring technique for cerebral perfusion [15].

#### 2.1.2. Relevant Parameters

The normal range of SjvO_2_ is approximately 55–75% [9]. Previous studies have identified the following influencing factors of SjvO_2_: CBF, cerebral metabolic rate of oxygen (CMRO_2_), arterial oxygen content (CaO_2_), and Hb [16]. When the cerebral oxygen consumption is larger than the oxygen supply, the brain tissues will remove more oxygen from the blood, resulting in a decrease in SjvO_2_. When CBF falls below the threshold, the brain can no longer compensate for the influence of low CBF on brain metabolism by simply increasing oxygen uptake from the blood. As a result, the brain tissue oxygen consumption gradually decreases, which is accompanied by a transition from aerobic to anaerobic metabolism. At the same time, the lactic acid content of the blood returning from the body to the heart through the superior (SVC) and inferior vena cava (IVC) increases [17,18]. When the brain oxygen supply is larger than the oxygen consumption, SjvO_2_ will increase.

For a healthy brain, CMRO_2_ and CBF are often correlated [16]. According to Fick’s law, CMRO_2_ can be calculated as

(1) $${\mathrm{CMRO}}_{2}=\mathrm{CBF}\times \left({\mathrm{CaO}}_{2}-{\mathrm{CjvO}}_{2}\right)$$

where

(2) $${\mathrm{CaO}}_{2}=\left(\mathrm{Hb}\times 1.34\times {\mathrm{SaO}}_{2}+0.003\times {\mathrm{PaO}}_{2}\right)$$

where ${\mathrm{SaO}}_{2}$ is the arterial oxygen saturation; ${\mathrm{PaO}}_{2}$ is the arterial partial pressure of oxygen; and

(3) $${\mathrm{CjvO}}_{2}=\left(\mathrm{Hb}\times 1.34\times {\mathrm{SjvO}}_{2}+0.003\times {\mathrm{PjvO}}_{2}\right)$$

where ${\mathrm{CjvO}}_{2}$ is the jugular venous oxygen content;$\;{\mathrm{PjvO}}_{2}$ is the jugular venous partial pressure of oxygen.

Since the dissolved oxygen is negligible and the amount of total hemoglobin is basically stable, the oxygen content in the brain is proportional to the cerebral blood oxygen saturation. Therefore, the arteriovenous difference of oxygen (AVDO_2_) can be determined by the difference between arterial oxygen saturation (SaO_2_) and SjvO_2_, and the Formula (1) can be converted to

(4) $${\mathrm{AVDO}}_{2}={\mathrm{CMRO}}_{2}/\mathrm{CBF}$$

It is obvious that SjvO_2_ is a physiological parameter related to SaO_2_, CBF, and CMRO_2_. When CMRO_2_ rises but CBF does not increase, AVDO_2_ will increase due to cerebral oxygen extraction, resulting in a decrease in SjvO_2_.

#### 2.1.3. Current Research Status

SjvO_2_ monitoring method is widely used in the clinic. It was initially used for neurocritical care and later for cardiac surgery and neurosurgery. SjvO_2_ is commonly applied to track compromised cerebral perfusion in traumatic brain injury (TBI) and subarachnoid hemorrhage (SAH), in addition to guiding the maintenance of cerebral perfusion pressure and treatment of hyperventilation [19]. 

Most TBI patients present with pathological manifestations of hypoxic brain injury. Maintaining the cerebral blood oxygen level within a normal range is extremely important for the prognosis of patients. Juan [20] and his team analyzed 1463 TBI patients in Spain and found that most patients received ICP monitoring but very few received SjvO_2_ monitoring. However, some researchers recommend early SjvO_2_ monitoring to uncover early changes in CBF in a timely manner [21]. Fandino [22] et al. also endorsed the importance of early SjvO_2_ monitoring. Multimodal monitoring of 50 TBI patients who had just been rescued showed that the level of SjvO_2_ desaturation was the only factor significantly related to prognosis. Sharf [23] et al. also demonstrated the importance of SjvO_2_ monitoring in TBI patients. Moreover, Vigué [24] et al. carried out brain monitoring of 27 TBI patients who had just been resuscitated. Their CPP was elevated to 70 mmHg as deemed appropriate and monitoring of pre-treatment mean arterial pressure (MAP), ICP, CPP, and arterial and venous blood gas was performed. A good correlation was found between CPP, MAP, and SjvO_2_. Early SjvO_2_ monitoring can compensate for the defects of MAP monitoring and facilitate the identification of patients with low CPP and high-risk cerebral ischemia. Apparently, SjvO_2_ monitoring is a beneficial complement to conventional ICP and CPP monitoring and is applicable to early monitoring of TBI patients. SjvO_2_ monitoring is conducive to the early recognition of cerebral ischemia and hypoxia and is closely related to patient prognosis.

Although SjvO_2_ monitoring is a commonly used method during brain surgery in adults, reports of this technique being used in children are limited. Sharma [25] et al. analyzed the clinical data of 19 children receiving brain surgery, and 11 of the children had experienced at least one episode of cerebral hypoxia. None of these children encountered complications during surgery in the presence of SjvO_2_ monitoring; therefore, it was concluded that SjvO_2_ monitoring can optimize and individualize the choice of hemodynamic and ventilation parameters among different types of pediatric cases receiving brain surgery.

In addition, SjvO_2_ monitoring can also be applied to stroke patients. Guven [26] and his team studied 82 high-risk stroke patients by monitoring and recording their physiological parameters and followed up. It was found that SjvO_2_ monitoring is conducive to recognizing high-risk stroke symptoms and guiding intervention in the critically ill.

SjvO_2_ monitoring is also a common choice during cardiopulmonary bypass (CPB). Kadoi [27] et al. carried out subgroup analysis of 185 patients receiving elective coronary artery bypass grafting (CABG). Their SjvO_2_ was continuously monitored and the corresponding hemodynamic parameters and arterial and venous blood gas values were recorded at specific intervals. It was found that early cognitive impairment in the old is related to an intraoperative reduction in SjvO_2_ during normal temperature extracorporeal circulation and requires extra attention. Buunk [28] and his team studied the prognostic significance of mixed venous oxygen saturation (SmvO_2_) and SjvO_2_ in patients with sudden cardiac arrest. Monitoring of 30 coma patients who had been recovered from cardiac arrest was performed and a more significant increase in SjvO_2_ than in SmvO_2_ was recorded in 12/21 of the dead subjects. In contrast, this phenomenon was only observed in 1/9 of the living patients; therefore, SjvO_2_/SmvO_2_ is an indicator of adverse outcome in such patients. Nevertheless, the separate role of SjvO_2_ needs to be clarified by subsequent studies. Souter [29] et al. performed postoperative monitoring of 22 patients receiving CABG and recorded the changes in their SjvO_2_. Among them, 15 patients underwent brain desaturation, which at an early stage after surgery, usually indicates insufficient cerebral perfusion. This observation is probably related to early brain injury. SjvO_2_ monitoring is a helpful method for patients receiving CPB, since it can reflect physiological background changes, assist with emergency rescue, improve prognosis, and even reduce mortality.

**Figure 2:**
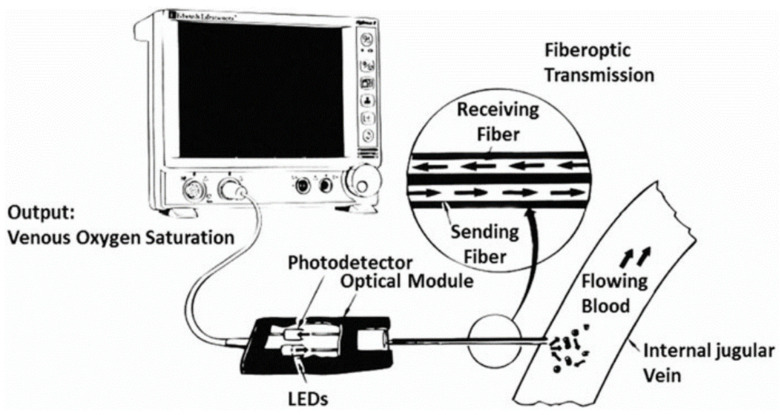
Working principle of the SjvO_2_ monitoring equipment [18].

SjvO_2_ monitoring equipment has evolved along with technical progress. In the early 20th century, SjvO_2_ measurements were initially obtained by direct puncture of the jugular bulb. Later, long-term tubes appeared, which were inserted at a high position in the jugular vein. In this way, repeated blood sampling for SjvO_2_ monitoring is achieved without the need for repeated punctures. In recent years, optical fiber technology has fueled the development of in vivo spectrophotometric catheters. At present, the two most commonly used types of equipment are the Abbott system and the Baxter-Edwards system [30]. The working principles of these two types of equipment are illustrated in Figure 2. Each is composed of a processor, an optical module, and a fiber catheter. During the measurement process, light composed of two or more wavelengths is delivered to the blood at the monitoring site via an optical fiber inside the catheter. The light is reflected by the red blood cells to a photoelectric sensor in another optical fiber. In the context of SjvO_2_ monitoring, light absorption varies under different concentrations of HbO_2_ in the blood. These two types of equipment evaluate SjvO_2_ based on analysis of the reflected light.

The Edslab uses two wavelengths of light for reflectance spectroscopy and performs calibration based on patient blood samples [15,16]. The Oximetrix 3 system uses light composed of three wavelengths for SjvO_2_ monitoring and can simultaneously measure the Hb concentration and blood oxygen saturation. It is capable of in vivo and in vitro calibration and reduces artifacts to a minimum [9,30]; however, some researchers suggest in vivo calibration at least once daily and in the case of reading errors [11,16].

#### 2.1.4. Analysis of the Advantages and Disadvantages

The reason why SjvO_2_ monitoring is superior than other methods is that it can monitor whole-brain blood oxygen saturation and capture the variation trend in real-time with a specific rise during cerebral ischemia, but this method is invasive. The longer the monitoring time, the higher the risks of hematoma and venous thrombosis. Additionally, since SjvO_2_ monitoring evaluates the whole-brain blood oxygen level, it is less sensitive to regional cerebral ischemia and hypoxia. Thus, a normal SjvO_2_ value does not necessarily indicate an absence of regional cerebral ischemia. However, a low SjvO_2_ value always indicates a reduction in CBF. Moreover, neither blood samples from the left nor right jugular vein can precisely represent the cerebral venous blood, and a difference in the SjvO_2_ values monitored at each side definitely exists [19].

### 2.2. Monitoring of Brain Tissue Partial Pressure of Oxygen

PbtO_2_ monitoring is an emerging cerebral blood oxygen saturation monitoring technique that has developed along with advances in electronics and optical fiber technology. PbtO_2_ can reflect the oxygenation of brain tissues at the cellular level as well as the perfusion and circulation statuses [31]. This method monitors brain tissue partial pressure of oxygen, temperature, and pH using implanted microelectrodes [8]. In this way, the regional energy metabolism and substance circulation in brain tissues can be captured and a better balance between oxygen consumption and supply is achieved. Moreover, PbtO_2_ monitoring can detect whether brain tissues have undergone irreversible injury due to ischemia and hypoxia.

#### 2.2.1. Working Principles

PbtO_2_ values reflect the balance between oxygen supply and consumption by brain tissues over a certain period of time. If the oxygen consumption is maintained at a constant level, PbtO_2_ values can better reflect the oxygen supply to brain tissues than can conventional indicators [32]. PbtO_2_ monitoring is typically conducted using a polarographic microcatheter equipped with an oxygen sensor [33]. After calibration with liquid nitrogen (partial oxygen pressure 0 kPa) and air (partial oxygen pressure 20.48 kPa), the microcatheter is inserted into the target brain tissues to directly measure the dynamic changes in local PbtO_2_ values.

Kautsky discovered the dynamic fluorescence quenching effect of oxygen molecules in 1939 [34], and this new technology has been increasingly applied to monitor brain tissue partial pressure of oxygen with a reduced cost. A fiber optic sensor measures brain tissue partial pressure of oxygen based on the fluorescence intensity of ruthenium complexes. The fluorescence intensity at the monitoring site is related to the brain tissue partial pressure of oxygen and can be expressed by the Stern–Volmer equation

(5) $$\frac{I_{0}}{I_{p}}=1+{\mathrm{kPO}}_{2}$$

where I_0_ is the fluorescence intensity under a partial oxygen pressure of 0; I_p_ is the fluorescence intensity under a partial oxygen pressure of P; and k is a constant.

#### 2.2.2. Relevant Parameters

Different normal ranges of PbtO_2_ values and the thresholds of ischemia have been provided by various researchers. Tisdall [35] et al. showed that the normal range of PbtO_2_ values is 35–50 mmHg and the recommended threshold of ischaemia is 5–20 mmHg. Schmidt [36] et al. suggested that the critical threshold for PbtO_2_values is 15–20 mmHg; PbtO_2_ values below this threshold may predict an adverse outcome such as cerebral infarction. Quanhong [37] et al. demonstrated that the normal range of PbtO_2_ values is 25–30 mmHg in general situations, and Jaeger [38] et al. arrived at a similar result. However, Yanlong [39] et al. highlighted that the normal range of PbtO_2_ values should be 15–40 mmHg. Cerebral ischemia and hypoxia are indicated at PbtO_2_ < 15 mmHg. As can be seen, there is still no generally accepted range of normal PbtO_2_ values to date. It is typically believed that a PbtO_2_ value < 15 mmHg must be handled immediately. During actual surgeries, PbtO_2_ is often used concomitantly with other indicators for brain oxygenation monitoring [40].

#### 2.2.3. Current Research Status

PbtO_2_ monitoring was first applied to the management of severe TBI patients in ICU but is now widely used in ICU bedside monitoring and perioperative anesthetic management [41]. PbtO_2_ is a complex dynamic variable reflecting the balance between brain oxygen supply and demand. A reduction in PbtO_2_ is considered, at least in theory, to be caused by oxygen deficiency or increased brain oxygen metabolism. An increase in PbtO_2_ is usually caused by an imbalance between CBF and brain oxygen metabolism or failure of the automated oxygen regulatory mechanism [42]. 

Quanhong [37] et al. showed that PbtO_2_ monitoring can accurately reflect brain metabolism and allow for the discovery of transient, early brain hypoxia, thus providing a reference for early treatment and diagnosis. Zauner [43] et al. indicated that the prognosis of patients with severe brain trauma is significantly positively correlated with PbtO_2_ values. Similarly, Kiening [44] and his team performed simultaneous SjvO_2_ and PbtO_2_ monitoring in 15 patients with severe brain trauma, and they found that the PbtO_2_ data were of higher quality and more suitable for long-term monitoring. PbtO_2_ monitoring is more helpful in reducing secondary poor brain oxygenation following severe brain trauma, so is more conducive to improving prognosis. Hongtao [45] and his team administered mild hypothermia treatment (MHT) in 68 severe TBI patients and monitored the changes in PbtO_2_, ICP, SjvO_2_, and CPP. It was found that during MHT, PbtO_2_, SjvO_2_, and CPP increased dramatically, while ICP decreased considerably. However, PbtO_2_values decreased after MHT. It was concluded that PbtO_2_ values can help to monitor regional brain oxygen metabolism, thereby guiding clinical treatment and prognostic prediction. Doppenberg [46] et al. carried out PbtO_2_ monitoring in 25 patients with acute brain injury. Xenon CT was performed to scan regional CBF (rCBF) near the target site and it was found that rCBF was significantly positively correlated with PbtO_2_. This technique is capable of continuously monitoring substrate release. PbtO_2_ monitoring not only detects cerebral ischemia and hypoxia but also reflects other physiological background changes and can help medical workers to further understand the condition of the patient.

PbtO_2_ monitoring is considered as a reliable bedside technique. Many studies have indicated that PbtO_2_ monitoring is a useful complement to conventional techniques. Qiang [47] et al. showed that in comparison with standard ICP/CPP-guided treatment, PbtO_2_ monitoring in conjunction with ICP/CPP-guided treatment can improve the outcome of TBI patients. Pierre [48] et al. also indicated that PbtO_2_ monitoring can help to control CPP and prevent secondary cerebral ischemia as a complement to standard ICP monitoring.

PbtO_2_ monitoring is rarely reported in pediatric cases either. Anthony [49] et al. carried out hypoxic challenge tests and baseline transcranial Doppler in 28 children with severe TBI, and post-test monitoring of PaO_2_, PbtO_2_, and CaO_2_ was performed. It was found that hyperoxia under normal pressure increases PbtO_2_; the more significant the response of PbtO_2_, the worse the prognosis. Some scholars are concerned with improving brain oxygenation based on PbtO_2_ changes. Sérgio [50] et al. studied 20 TBI patients with acute respiratory distress syndrome, who were successively given positive end-expiratory pressure of 5, 10, and 15 cmH_2_O. PbtO_2_, SO_2_, CPP, and ICP were recorded within 20 min and it was found that elevating the positive end-expiratory pressure increased the PbtO_2_ values. In these patients, brain oxygenation could be improved by raising the positive end-expiratory pressure.

PbtO_2_ monitoring has also been used in endoscopic endonasal skull base surgery. Tomasz [51] et al. carried out intraoperative monitoring of ICP, CPP, and arterial pressure (AP) in conjunction with simultaneous PbtO_2_ monitoring in five patients with confirmed basicranial tumor and arterial hypertension. It was found that in patients with extremely low PbtO_2_ values, AP should be appropriately lowered in order to obtain an area that does not bleed during surgery. 

During surgery for SAH, a lower PbtO_2_ value can indicate postoperative vasospasm and predict a poor prognosis. Since PbtO_2_ monitoring intuitively reflects oxygenation in the target area, it is also used for endovascular treatment in SAH and vasospasm patients [52]. 

**Figure 3:**
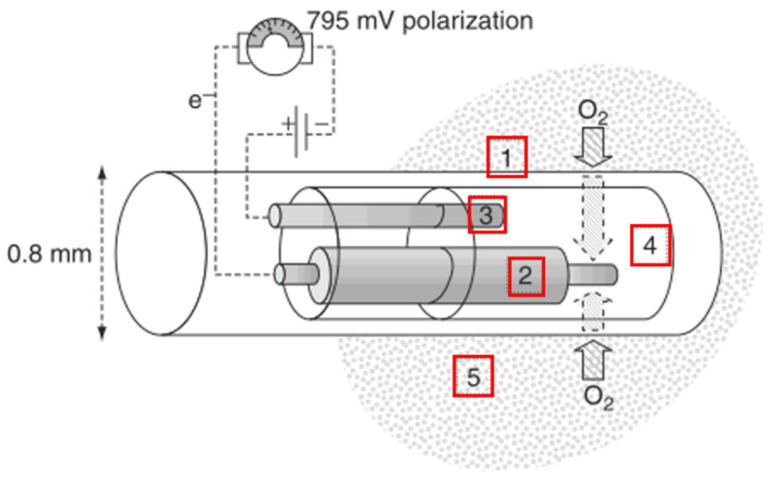
Schematic diagram of the electrodes in the Licox system 1. Polyethylene tube with diffuser film; 2. Gold polarographic cathode; 3. Silver polarographic anode; 4. Electrolysis chamber; 5. Target monitoring area of the brain [31].

In recent years, the mature PbtO_2_ monitoring equipment has divided into two major categories and adopted the two working principles described above. That is, the PbtO_2_ monitoring equipment based on a Clark-type oxygen electrode and the fluorescence-based equipment. The former is usually composed of silver-, gold-, or platinum-reducing oxygen. Changes in the voltage between the reference electrode (anode) and the measuring electrode (cathode) are directly proportional to the amount of molecular oxygen reduced at the cathode. In the Clark-type oxygen electrode, both the anode and cathode are placed behind a film possessing oxygen permeability and electrical insulation properties [8]. The Licox system is the most common equipment based on the Clark-type oxygen electrode, as shown in Figure 3, and offers brain oxygen monitoring of a 7.1–15 mm^2^ PbtO_2_-sensitive area. The oxygen diffusing from the brain tissues through the semipermeable film is reduced by the gold polarographic cathode, generating a current that is directly proportional to the oxygen concentration [31]. Since the Licox system lacks an integrated temperature sensor, it is necessary to insert an extra temperature monitoring module into the brain to correct the PbtO_2_ values in response to brain temperature changes [33]. This equipment is highly suitable for long-term use and has already been implanted during surgery [8].

**Figure 4:**
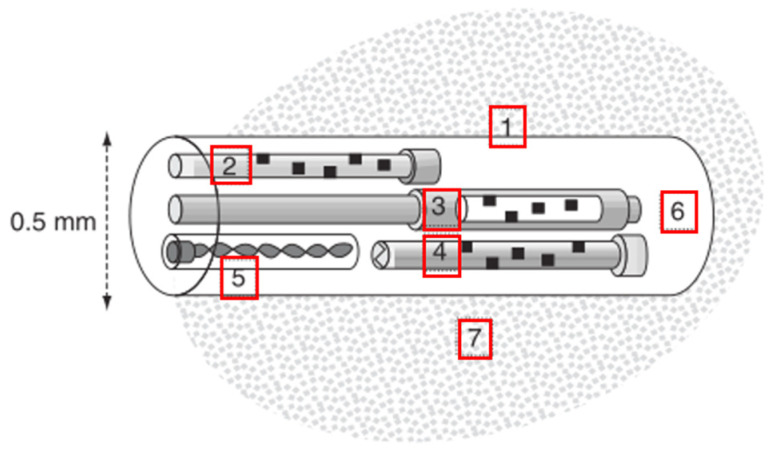
Schematic diagram of the sensor in the NeuroTrend system 1. Microporous polyethylene tube; 2. PbtO_2_ sensor (ruthenium dye in an organosilicon matrix); 3. PbCO_2_ sensor (phenol red in a supercarbonate solution); 4. pH sensor (phenol red in a polyacrylamide gel); 5. Thermocouple (copper wire and constantan wire); 6. Phenol red and polyacrylamide gel; and 7. Target monitoring area of the brain [31].

The NeuroTrend system is the most common fluorescence-based equipment. Its precursor is the Paratrend system, an intra-arterial monitoring system. After modification, it can be used for cerebral blood oxygen saturation monitoring [33]. The NeuroTrendsystem has a photodiode as its data acquisition front-end (Figure 4), which is installed at the top of a flexible tube. This system can be used for monitoring PbtO_2_, PbCO_2_, pH, and temperature. The NeuroTrend system measures the brain substance concentration according to changes in the optical properties of the indicator caused by photochemical reactions. For example, in the presence of oxygen, the intensity of fluorescence emitted by the indicator ruthenium is attenuated, a phenomenon that can be utilized for PbtO_2_monitoring. Unlike the Licox system, this process in the NeuroTrend system does not consume oxygen or influence the monitored oxygen level. The local pH affects the intensity of light passing through phenol red; thus, the pH sensor achieves monitoring through light absorption. Likewise, the PbCO_2_ monitoring equipment is also a CO_2_-selective pH sensor. In contrast, local temperature monitoring is realized through a thermocouple [31]. This equipment has been proven, by long-term clinical application, to be a qualified alternative to conventional in vitro arterial blood gas analysis [8].

#### 2.2.4. Analysis of the Advantages and Disadvantages

PbtO_2_ monitoring has unique advantages in comparison with other methods: (1) ease of operation and high data reliability without an apparent drift in the monitoring values, and hence no need for frequent corrections; (2) a better reflection of the oxygen supply and consumption by brain tissues and timely discovery of irreversible injury to brain tissues caused by ischemia and hypoxia, with higher sensitivity than other cerebral blood oxygen saturation monitoring methods; and (3) a more helpful indicator of brain death since PbtO_2_ rapidly drops to 0 kPa during the early stages of brain death. However, PbtO_2_ monitoring also has disadvantages: (1) if the microelectrodes are placed in an area with a brain injury, the real situation of whole-brain oxygen metabolism may be concealed by regional cerebral ischemia and hypoxia, leading to false estimation; (2) it is an invasive technique that requires placement of microelectrodes in the human brain; therefore, this method is likely to cause local injury of brain tissues and increase the risk of intracranial infection; and (3) in clinical practice, it is time-costly to achieve temperature balance after electrode placement, and effective data can only be acquired subsequently. Dings [53] et al. carried out monitoring in 73 patients with brain trauma, with the longest monitoring duration being 16 days. None of the patients had any intracranial infection symptoms, and the incidence of intracranial hematoma was less than 2.7%. Moreover, none of these patients needed surgical treatment. Based on this, many researchers believe that PbtO_2_ monitoring is safe and accurate so that is ideal for cerebral blood oxygen saturation monitoring.

### 2.3. NIRS-Based Monitoring

NIRS is a new optical technique measuring blood oxygenation of the brain and detecting brain activities that emerged at the end of the last century. NIRS is a promising and reliable method for understanding human activities and is generally applied to the diagnosis and examination of brain diseases [54,55]. In 1977, Jobsis published his research findings in Science, proposing the utilization of the tissue permeability property of near-infrared light for the first time. A sensor installed in the scalp was able to noninvasively capture information deep within thick tissues such as the brain. Soon afterward, many researchers began to use optical methods to study human brain activities. NIRS not only assists in the in-depth analysis of how the brain processes the information but also reveals many other brain functions. In other words, NIRS can help diagnosing and treating various brain diseases and is the only non-invasive bedside cerebral blood oxygen saturation monitoring technique available so far, which has been increasingly applied in recent years.

#### 2.3.1. Working Principles

NIRS-based detection of cerebral blood oxygen saturation is based on the different spectral absorption features of oxyhemoglobin (HbO_2_) and reduced hemoglobin (HbR) in the tissues detected within the near-infrared optical window (700–950 nm). Appropriate wavelengths are chosen to estimate cerebral blood oxygen saturation from the light intensity attenuation at two or more wavelengths. Variations in the HbR and HbO_2_ content are calculated according to the absorption law [56]. Finally, the cerebral blood oxygen saturation values are estimated using the predictive model.

The modified Lambert–Beer law lays an essential theoretical foundation for NIRS-based detection. Human tissues can weaken the incident light intensity through absorption, scattering, and reflection. Due to the strong scattering effect of human tissues, the intensity of the scattered light is far greater than that of the absorbed light [57]. According to the Lambert–Beer law, the transmission path of photons is the straight-line distance between the detector and the light source. However, in actual monitoring, the transmission path length of photons is far greater than the straight-line distance between the detector and the light source due to the scattering effect of human tissues. To address this problem, Cope and Delpy [58] proposed the modified Lambert–Beer law, which considers multiple scattering events and attenuation of light in human tissues. The transmission process of light is described by the average and differential optical path lengths

(6) $$\mathrm{OD}=\log\left(\frac{I_{0}}{I_{t}}\right)=\mathrm{DPF}\cdot \rho \underset{i}{\sum }\varepsilon_{i}\cdot c_{i}+G$$

where OD is the optical density, also known as absorbance; I_0_ is the incident light intensity; I_t_ is the emergent light intensity; c_i_ is the concentration of the detected substance; $\varepsilon_{i}$ is the molar absorption coefficient, which depends on the wavelength and properties of the detected substance; G is defined as the constant attenuation factor caused by background scattering or absorption; and DPF is the differential path factor, defined as the ratio of the actual optical path length in tissues to the distance between each detector and the light source. DPF can be calculated by Monte Carlo simulation or using time-resolved or frequency domain time-resolved technology.

#### 2.3.2. Relevant Parameters

Changes in cerebral blood oxygen saturation are closely related to the cerebral circulation. Either disease or physiological activities influence the cerebral circulation and consequently alter the cerebral blood oxygen saturation values. Cerebral blood oxygen saturation monitoring is a way to observe lesions as well as evaluate the physiology of patients, which is why cerebral blood oxygen saturation serves as an important physiological indicator.

The formula for cerebral blood oxygen saturation varies among researchers and products; some express it as SctO_2_, StO_2_, or rStO_2_, and others express it as rSO_2_. In theory, rSO_2_ is the weighted average of cerebral arterial, capillary, and venous oxygen saturation [59,60]. In actual monitoring, rSO_2_ is generally defined as the percentage of oxygen carried by hemoglobin at the target monitoring site, the basic formula for which is

(7) $${\mathrm{rSO}}_{2}=\frac{C_{{\mathrm{HbO}}_{2}}}{C_{{\mathrm{HbO}}_{2}}+C_{\mathrm{HbR}}}\times 100\%$$

where $C_{{\mathrm{HbO}}_{2}}$ is the oxyhaemoglobin concentration; and $C_{\mathrm{HbR}}$ is the reduced hemoglobin concentration, which refers to the content of the corresponding type of hemoglobin per unit volume of blood. The results are usually expressed as a percentage between 0% and 100%.

Disease deterioration and improvement can be intuitively reflected by changes in rSO_2_. Moreover, rSO_2_ is a crucial indicator for guiding surgical anesthesia in the clinic. rSO_2_ monitoring can help to prevent damage to human tissues and reduce the incidence of complications due to long-term low cerebral oxygenation.

#### 2.3.3. Current Research Status

In recent years, more studies on the NIRS-based monitoring of cerebral blood oxygen saturation were published. The use of NIRS monitoring during the perioperative period has also increased significantly, especially in cardiac surgery, carotid surgery, and surgery performed in the beach chair position [61]. 

Heringlake [62] et al. performed a prospective observational study in 1178 patients receiving cardiac surgery with extracorporeal circulation. It was found that preoperative rSO_2_, instead of organ perfusion, is a useful complementary tool for risk stratification of these patients. However, rSO_2_ may be influenced by body temperature and perfusion rate in patients receiving extracorporeal circulation. Yichao [63] et al. performed rSO_2_ monitoring in 15 patients receiving extracorporeal circulation using a self-developed near-infrared cerebral oxygenation monitor. It was found that rSO_2_ values are negatively correlated with body temperature and positively correlated with perfusion rate. The rSO_2_ values are lower under a lower perfusion rate and during the early stage of rewarming. NIRS-based monitoring of rSO_2_ during extracorporeal circulation is thought to protect brain tissues and uncover ischemia and hypoxia. Parnia [64] et al. employed NIRS to monitor rSO_2_ in 183 patients who had experienced in-hospital cardiac arrest (IHCA). It was found that rSO_2_ can be used to evaluate cerebral oxygenation during cardiopulmonary resuscitation, and an improved rSO_2_ predicts a better survival rate and spontaneous circulation recovery after IHCA. Wally [65] et al. showed that NIRS monitoring during cardiac surgery significantly reduces the number and severity of perioperative complications and shortens the duration of intensive care. As shown above, NIRS monitoring during cardiac surgery can reflect the cerebral oxygenation level in an effective, real-time manner, providing valuable information for diagnosis and treatment and reducing secondary injury caused by ischemia and hypoxia.

Among the researches on brain injury, Dunham [66] et al. performed an observational study in 18 TBI patients and found that rSO_2_ values under 60% are correlated with mortality, intracranial hypertension, and decreased cerebral perfusion pressure. Yokose [67] et al. applied time-resolved spectral monitoring in 14 SAH patients, most of whom were graded as poor, and reported that a 3.9–6.4% reduction in rSO_2_ is the optimal threshold for recognizing ischemia.

At present, there is still no gold standard for NIRS monitoring. Moreover, there is no consensus regarding the normal range of rSO_2_ values or critical thresholds for ischemia and hypoxia. Different judgment criteria have been proposed. Samra [68] et al. analyzed 94 patients receiving carotid endarterectomy (CEA) under local anesthesia. It was found that rSO_2_ values decrease more significantly in cerebral ischemia patients with neuropathy, and after logistic regression it was concluded that a 20% reduction in rSO_2_ from the baseline can be used as the trigger point for improving cerebral oxygenation. Moritz [69] et al. suggested using 59% rSO_2_ as the clinical threshold for recognizing ischemia. However, Kirkpatrick [70] indicated that the threshold for cerebral ischemia determined for local anesthesia is not fit for general anesthesia, and vice versa.

In addition, NIRS is also used to monitor cerebral oxygenation during other types of surgery. Sørensen [71] et al. found that during liver transplantation, NIRS-based rSO_2_ monitoring can uncover impairments in the automated oxygen regulatory mechanism of the brain, cerebral hypoxia during the anhepatic phase, and cerebral hyperoxemia during post-transplant perfusion. This method is of high importance for the prediction and discovery of neurological complications related to liver transplantation. Henning [72] showed that after chest surgery, large-scale orthopedic surgery, and abdominal surgery, postoperative cognitive dysfunction (POCD) may be related to intraoperative cerebral desaturation. Tamara [73] et al. divided 43 patients indicated for surgical treatment of lumbar diseases into two groups. One group received intraoperative NIRS monitoring and the same neurocognitive testing before surgery and at 7 days and 1 month after surgery. There was a significant difference in the incidence of cognitive defects between the subgroups with and without NIRS monitoring. It was concluded that NIRS is conducive to a reduction in the incidence of POCD in patients receiving surgery in the prone position. Viola [74] et al. employed NIRS and TCD to study 21 patients with amnestic mild cognitive impairment (aMCI) and 10 healthy controls. It was found that a reduction in the tissue oxygenation index (TOI) in the bilateral temporoparietal cortex and an increase in the middle cerebral artery pulsatility index (PI) are correlated with the clinical diagnosis of aMCI; thus, TOI reduction can be used as a novel indicator of aMCI. Although many studies have demonstrated that POCD may be closely related to intraoperative NIRS monitoring results, further clinical investigations are needed to understand the relevant working mechanism.

NIRS monitoring has also been applied to cerebral blood oxygen saturation monitoring in infants and young children, and does not usually cause extra damage in these patients thanks to its non-invasive and safe nature. It is also considered as the most suitable cerebral blood oxygen saturation monitoring method for infants and young children [75]. Tran [76] et al. found that for infants under 1 year old, there exists significant differences in cerebral blood oxygen saturation at different body positions. rSO_2_ monitoring is an effective way for physicians to understand infant brain health conditions with a view to adjust the nursing procedures and protect the brain. Chock [77] et al. performed NIRS-based rSO_2_ monitoring in 103 premature infants within 96 h of birth. It was demonstrated that early NIRS monitoring is able to detect a reduction in rSO_2_ and changes in the automated oxygen regulation of the brain, thereby providing a basis for determining the risk of death or neuroradiographic injury. Ozawa [78] et al. carried out rSO_2_ monitoring in 127 healthy full-term infants within 10 min of birth and determined the reference range for rSO_2_. Berens [79] et al. conducted real-time tracking of rSO_2_ changes after aortic occlusion during aortic coarctation repair in 26 patients. Comparative analysis revealed that rSO_2_ monitoring can provide real-time trend information regarding cerebral oxygenation under aortic occlusion. rSO_2_ monitoring is highly important for understanding the health conditions of infants and young children [80]. Early treatment should be administered if cerebral ischemia and hypoxia are discovered so as to prevent further aggravation and unnecessary burden.

Along with the research progress in NIRS-based rSO_2_ monitoring, many new types of monitoring equipment emerged, most of which are based on the above-mentioned principles. Although each has its own special technical features, NIRS-based rSO_2_ monitoring equipment mainly utilizes three types of method: continuous wave spectroscopy (CWS), time-resolved spectroscopy (TRS), and frequency domain photon migration (FDPM) [10,55]. CWS is the most simple and reliable in terms of system function realization. CWS is featured by a short duration of data acquisition and the capability of monitoring relative changes in rSO_2_ values but not absolute changes. TRS can obtain the length of the light transmission path and the distribution of emergent light intensity over time, and hence the absorption and scattering coefficients of brain tissues. Accordingly, TRS can calculate the absolute concentration values of the two types of hemoglobin and the cerebral blood oxygen level; however, TRS requires the use of costly equipment, has a small dynamic range, and cannot use digital technology to increase the signal-to-noise ratio (SNR). FDPM can also obtain the light transmission path length and the photon migration time from phase delay. Accordingly, different optical parameters of brain tissues and the absolute cerebral blood oxygen level are calculated. This method has a relatively shorter data reading time but places a higher requirement on hardware and is much more difficult to realize than CWS. Due to the difficulty of technological realization, a lot of rSO_2_ monitoring equipment on the market has been developed based on CWS. The other two methods are currently under scientific investigation and have not yet entered the stage of clinical application. In addition to the above three techniques, functional NIRS (fNIRS) has also emerged in recent years. This technique can generate patient head images of a larger area and higher temporal resolution, thereby visualizing the oxygenation changes in brain tissues. fNIRS is also known as diffuse optical imaging (DOI) or diffuse optical tomography (DOT); however, this method is usually inapplicable to clinical monitoring due to its high cost.

**Figure 5:**
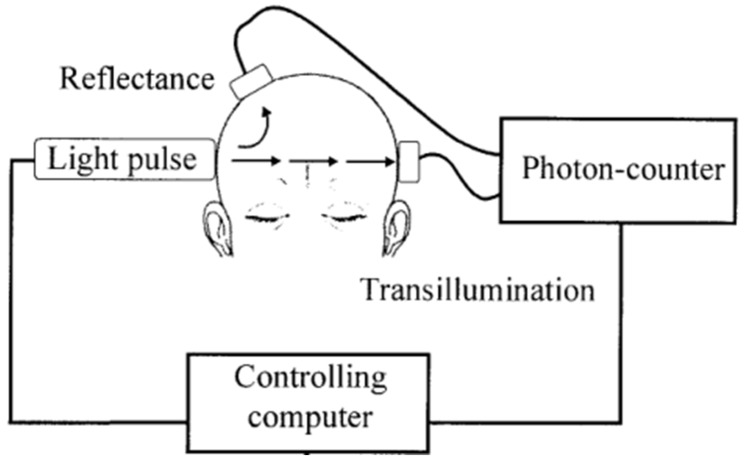
The basic structure of NIRS-based equipment for rSO_2_ monitoring [81].

**Figure 6:**
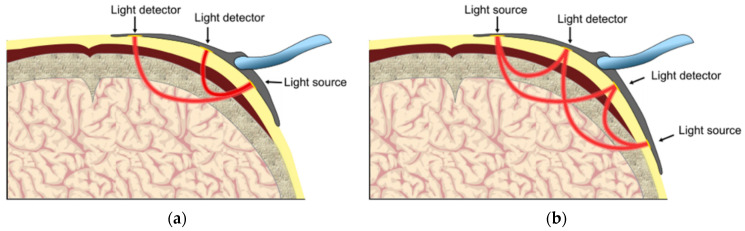
Diagram of the NIRS sensor monitoring light path [10]. (**a**) Single light source; (**b**) dual light source.

The basic structure of NIRS-based equipment for rSO_2_ monitoring are shown in Figure 5. The NIRS non-invasive monitoring sensors of this equipment are placed in the target monitoring areas, typically the forehead. An NIR module is usually composed of two parts, a light source and a photoelectric detector. The light source emits infrared light at certain wavelengths that penetrate the tissues, and the photoelectric detector measures the emergent light intensity, followed by conversion of the emergent light intensity into useful clinical information, as shown in Figure 6. Such equipment is further divided into transmission NIRS and reflectance NIRS depending on the position of the photoelectric detector. Transmission NIRS is usually used for cerebral oxygenation monitoring in infants and young children. Moreover, some equipment has several receiving light paths and is therefore known as multi-distance NIRS. This method can differentiate between light attenuation caused by the skull and the covering tissues and that caused by the brain tissues.

#### 2.3.4. Analysis of the Advantages and Disadvantages

NIRS-based monitoring of rSO_2_ has unique advantages: directly or indirectly detecting physiological changes and metabolic processes, it is easy to realize, and involves simple procedures. Moreover, the near-infrared light has a strong transmission capacity in human brain tissues. Within the optical window of 700–950 nm, the penetration depth of the near-infrared light can reach several centimeters cm [7,82]. In comparison with existing techniques—such as CT, fMRI, PET, and EEG—NIRS monitoring has a higher temporal resolution and is a non-invasive safe method for real-time continuous measurement [83]. Like any other technique, NIRS monitoring also has some drawbacks. For example, its signal-to-noise ratio (SNR) and spatial resolution are low, and in actual applications, selection of the monitoring site has a large influence on the results. Moreover, extracranial tissues can cause considerable interference with signals, and extracranial circulation may even contaminate the NIRS monitoring results. Different commercial monitoring equipment tends to use different algorithms; therefore, it is difficult to compare the monitoring results across equipment, and no standards have yet been established [84]. Furthermore, rSO_2_ presents extensive intra- and interindividual baseline variability. No definite normal range of rSO_2_ has been established and there is no gold standard to determine the thresholds for recognizing cerebral ischemia or hypoxia; therefore, NIRS-based rSO_2_monitoring equipment is generally only used for trend analysis.

## 3. Discussion

Primary brain injury, especially that caused by trauma or disease, cannot be reversed by treatment. Therefore, avoiding secondary brain injury caused by ischemia and hypoxia is the main goal of perioperative monitoring and postoperative nursing [85].

The cerebral blood oxygen saturation monitoring methods commonly used in research and in the clinic are provided in Table 1.

The usability, reliability, and safety of the monitoring methods have long been a focus for researchers. If the equipment and technique have ease of use, the risk of error will be relatively low. Moreover, such equipment or methods are more easily realized in clinical studies and treatments. Table 2 lists some features of these methods, and Table 3 enumerates the advantages and disadvantages of these methods in detail.

These three methods have been widely applied in the clinic. Apart from these, some techniques are primarily intended for research, such as PET and fMRI, which can be used to study the metabolism at specific sites and to compare across different sites. However, these techniques only provide snapshots but cannot be used as routine monitoring.

PET can be used to calculate local parameters—such as rCBV, rCBF, and OER—and is a highly reliable, non-invasive technique considered the gold standard for CBF measurement [86]. However, PET scanners are complex and bulky. Moreover, the short half-life of radioisotopes makes it almost impossible to scan and treat simultaneously, and PET is costly; therefore, it is more suitable for research purposes than clinical detection.

fMRI uses a blood oxygen level-dependent (BOLD) effect to evaluate the oxygenation at specific sites [87]. fMRI is another non-invasive technique that uses human tissues and cells as natural contrast agents, offering sufficiently high spatiotemporal resolution. Similar to PET, fMRI also has problems related to operational complexity and inconvenience and, more importantly, the unproven accuracy of detection [88]. Furthermore, due to the structural features and measurement requirements of the fMRI equipment, patients need to stay motionless; therefore, fMRI cannot be applied to children or patients with claustrophobia, and is mainly used for research purposes.

As mentioned above, these methods all have their own advantages and disadvantages. The current research results show that in the clinical perioperative period, the results obtained by invasive methods are relatively more accurate, and the PbtO_2_ monitoring method is better. Certainly, NIRS-based monitoring of rSO2 has become a popular topic over the past decade because of its advantages, as shown in Table 3. As a result of its non-invasiveness, ease of operation, and safety, this technique has been increasingly used by researchers during their investigations, yielding diverse findings.

However, its defects—including low SNR, low stability and reliability of monitoring data, and a higher probability of signals being contaminated—have restricted clinical application. Moreover, NIRS technology using two wave-lengths is more commonly employed in studies. Although this technology is easier to realize, its SNR is lower, resulting in poor reliability and stability in terms of the monitoring results. To overcome certain defects in NIRS-based monitoring of rSO_2_, researchers have proposed various solutions; for example, hardware system optimization, combination of several monitoring methods, optimization of the signal processing algorithms, and model optimization. It has been suggested that NIRS can be combined with spatially resolved spectroscopy to increase the spatial resolution and sensitivity of detection and reduce the influence of extracranial tissues [89]. Carlos [90] et al. realized the automated detection of noise channels for NIRS signals via correlation analysis, achieving high accuracy at a relatively lower computational cost. Kamran [91] et al. established an autoregressive–moving-average model (ARMAX), for which human physiological signals were used as the input. This method can reduce the interference in NIRS signals caused by human physiological noise and improve monitoring accuracy and reliability. Denault [92] et al. proposed an NIRS signal processing algorithm based on optimization factors to obtain reliable monitoring results. Jenny [93] et al. reduced the influence of individual differences by adjusting the geometric structure of the sensor, not only improving the measurement accuracy but also achieving a higher clinical correlation.

Furthermore, at the end of the 20th century, some researchers [58,94,95,96] discovered that when using optical technology to study the human brain, in addition to the absorption of light by hemoglobin in the blood, there are other strong absorption components, such as melanin, cytochrome, water, etc. In the modified Lambert–Beer law, the influence of these components is defined as a constant factor G, which will be eliminated when the result is calculated. However, in fact, this part of the impact cannot be removed directly by simple calculations. Instead, it is necessary to consider the specific circumstances of the physiological background through which light passes through the human brain tissue for analysis. Thus, a suitable mathematical model could be constructed to express the light intensity attenuation of the incident light after passing through the human brain tissue.

Although many companies have developed different equipment and put them into use, as an emerging cerebral blood oxygen saturation monitoring technology, it still takes a long time to address the defects.

## 4. Conclusions

Invasive techniques still prevail in clinical cerebral blood oxygen saturation monitoring and are considered to be accurate, but these methods may cause damage to the body and have other potential risks. Along with technological progress, non-invasive, painless medical procedures have gradually become the goal. A growing body of evidence shows that NIRS monitoring has bright application prospects. Although the application of NIRS monitoring techniques remains unsatisfactory, NIRS boasts many advantages such as non-invasiveness, ease of realization, low cost, simplicity of operation, and ability of continuous monitoring. All of these features coincide with the future development trend. NIRS is undoubtedly a powerful tool for cerebral blood oxygen saturation monitoring and can be used for both clinical and research purposes.

## Figures and Tables

**Table 1:** Overview of common methods for cerebral blood oxygen saturation monitoring.

Method	Contents Monitored	Depth of Monitoring	Indications
Jugular venous oxygen saturation monitoring	${\mathrm{SjvO}}_{2},\;{\mathrm{PjvO}}_{2}$,$\;{\mathrm{SaO}}_{2}$,$\;{\mathrm{PaO}}_{2}$, and Hb	250 μm [81]	TBI; SAH; neurosurgery; plastic surgery
Brain tissue partial pressure of oxygen monitoring	PbtO_2_, PCO_2_, and pH	Implantable electrode: 15–20 μm [81] Surface electrode: 20 μm [81]	TBI; SAH; continuous rSO_2_ monitoring; studies of cerebral oxygenation under different pathological conditions; neurosurgery
NIRS-based monitoring	rSO_2_, HbO_2_, HbR, CBF, and CBV	Maximum penetration depth: several centimeters [7,82]	Monitoring of Hb oxygenation in the shallow layer; carotid surgery; cardiac surgery; routine monitoring during general anesthesia; TBI

**Table 2:** Overall features of different methods.

Method	Invasiveness	Usability	Reliability	Safety
Jugular venous oxygen saturation monitoring	Minimally invasive	Ease of use	Intermediate ^1^	High
Brain tissue partial pressure of oxygen monitoring	Invasive	Ease of use	High	Good
NIRS-based monitoring	Non-invasive	Ease of use	Intermediate	High

^1^ Grading: High, good, intermediate, acceptable, poor.

**Table 3:** Summary of the advantages and disadvantages of different methods.

Method	Advantages	Disadvantages
Jugular venous oxygen saturation monitoring	Applicable to the evaluation of capillary Hb concentration; Wide application scope; Ease of use; Minimally invasive; Easy to pass through the auxiliary channel for standard diagnostic endoscope	An incomplete mix in the asymmetrical venous drainage of the brain may influence the evaluation of focal injury; Extracerebral contamination with blood from the scalp, meninges, and skull; Overestimation under alkaline conditions; Insensitive to the hypencephalon; Insensitive to regional ischemia and hypoxia of brain tissues
Brain tissue partial pressure of oxygen monitoring	PbtO_2_ is well correlated with PaO_2_; Ease of use; High data reliability; Provides reliable, consecutive cerebral blood oxygen saturation monitoring for several days; Capable of real-time monitoring; Selective simultaneous monitoring of PbtO_2_, PCO_2_, and pH; More able to determine brain death	Effective data acquisition does not begin until the temperature balance is reached before each measurement; Invasive; False monitoring results
NIRS-based monitoring	Low cost; Safe; Ease of use; High temporal resolution; Significantly reduces the incidence of organ dysfunction during cardiac surgery; Able to predict vasospasm; Capable of consecutive real-time monitoring; Assists in the diagnosis of epileptic seizures	The signals may be influenced by extracranial tissues; Poor temporal resolution; Baseline variability in rSO_2_ among individuals; Low SNR, Fuzziness of measurement results [54]; Difficulty in converting from research to bedside application; There is no impact on postoperative cognitive function, stroke, or mortality in patients receiving cardiac surgery; There is a lack of a gold standard for determining critical values and normal ranges

## Data Availability

Not applicable.

## References

[1] Rasulo F., Matta B., Varanini N. (2018). Cerebral Blood Flow Monitoring. Neuromonitoring Techniques.

[2] Zauner A., Daugherty W.P., Bullock M.R., Warner D.S. (2002). Brain Oxygenation and Energy Metabolism: Part I—Biological Function and Pathophysiology. Neurosurgery.

[3] Caine D., Watson J. (2000). Neuropsychological and neuropathological sequelae of cerebral anoxia: A critical review. J. Int. Neuropsychol. Soc..

[4] Roldán M., Kyriacou P. (2021). Near-Infrared Spectroscopy (NIRS) in Traumatic Brain Injury (TBI). Sensors.

[5] Sahinovic M.M., Vos J.J., Scheeren T.W.L. (2020). Journal of Clinical Monitoring and Computing 2019 end of year summary: Monitoring tissue oxygenation and perfusion and its autoregulation. J. Clin. Monit..

[6] Lewis C., Parulkar S.D., Bebawy J., Sherwani S., Hogue C.W. (2018). Cerebral Neuromonitoring During Cardiac Surgery: A Critical Appraisal with an Emphasis on Near-Infrared Spectroscopy. J. Cardiothorac. Vasc. Anesth..

[7] Bhatia A., Gupta A.K. (2007). Neuromonitoring in the intensive care unit. II. Cerebral oxygenation monitoring and microdialysis. Intensiv. Care Med..

[8] Hollinger A., Siegemund M., Cueni N., Steiner L.A. (2018). Brain Tissue Oxygenation. Neuromonitoring Techniques.

[9] Chaikittisilpa N., Vavilala M.S., Lele A.V. (2018). Jugular Venous Oximetry. Neuromonitoring Techniques.

[10] Denault A.Y., Shaaban-Ali M., Cournoyer A., Benkreira A., Mailhot T. (2018). Near-Infrared Spectroscopy. Neuromonitoring Techniques.

[11] White H., Baker A. (2002). Continuous jugular venous oximetry in the neurointensive care unit—a brief review. Can. J. Anaesth..

[12] Yang M.-T. (2020). Multimodal neurocritical monitoring. Biomed. J..

[13] Schell R.M., Cole D.J. (2000). Cerebral Monitoring: Jugular Venous Oximetry. Anesth. Analg..

[14] Richter J., Sklienka P., Setra A.E., Zahorec R., Das S., Chatterjee N. (2020). Is jugular bulb oximetry monitoring associated with outcome in out of hospital cardiac arrest patients?. J. Clin. Monit..

[15] Macmillan C.S.A., Andrews P.J.D. (2000). Cerebrovenous oxygen saturation monitoring: Practical considerations and clinical relevance. Intensiv. Care Med..

[16] Sharma D., Lele A. (2017). Monitoring of Jugular Venous Oxygen Saturation. Monitoring the Nervous System for Anesthesiologists and Other Health Care Professionals.

[17] Seubert C.N., Cibula J.E., Mahla M.E., Layon A.J. (2013). Noninvasive Monitoring in the Neurointensive Care Unit: EEG, Oximetry, TCD. Textbook of Neurointensive Care.

[18] Hiraki T., Ushijima K. (2015). Role of Jugular Venous Oxygen Saturation in Neuroanesthesia. Neuroanesthesia and Cerebrospinal Protection.

[19] Samra S.K., Rajajee V., Koht A., Sloan T.B., Toleikis J.R. (2012). Monitoring of Jugular Venous Oxygen Saturation. Monitoring the Nervous System for Anesthesiologists and Other Health Care Professionals.

[20] Llompart-Pou J.A., Barea-Mendoza J.A., Sánchez-Casado M., González-Robledo J., Mayor-García D.M., Montserrat-Ortiz N., Enríquez-Giraudo P., Cordero-Lorenzana M.L., Chico-Fernández M. (2020). Neuromonitoring in the severe traumatic brain injury. Spanish Trauma ICU Registry (RETRAUCI). Neurocirugía.

[21] Deyne C.D., Decruyenaere J., Calle P., Vandekerckhove T., Vaganee B., Garcia R.B., Colardyn F. (1996). Analysis of very early jugular bulb oximetry data after severe head injury: Implications for the emergency management?. Eur. J. Emerg. Med..

[22] Fandino J., Stocker R., Prokop S., Trentz O., Imhof H.-G. (2000). Cerebral oxygenation and systemic trauma related factors determining neurological outcome after brain injury. J. Clin. Neurosci..

[23] Sharf M.S., El-Gebali M.A. (2013). Correlation between Glasgow coma scale and Jugular venous oxygen saturation in severe traumatic brain injury. Egypt. J. Anaesth..

[24] Vigué B., Ract C., Benayed M., Zlotine N., Leblanc P.E., Bissonnette K.S. (1999). Early SjvO_2_ monitoring in patients with severe brain trauma. Intensive Care Med..

[25] Sharma D., Siriussawakul A., Dooney N., Hecker J.G., Vavilala M.S. (2013). Clinical experience with intraoperative jugular venous oximetry during pediatric intracranial neurosurgery. Pediatric Anesth..

[26] Guven M., Akilli N.B., Koylu R., Oner V., Guven M., Ozer M.R. (2020). A new marker identification of high risk stroke patients: Jugular saturation. Am. J. Emerg. Med..

[27] Kadoi Y., Saito S., Goto F., Fujita N. (2001). Decrease in jugular venous oxygen saturation during normothermic cardiopulmonary bypass predicts short-term postoperative neurologic dysfunction in elderly patients. J. Am. Coll. Cardiol..

[28] Buunk G., Van J.G., Meinders A.E. (1999). Prognostic significance of the difference between mixed venous and jugular bulb oxygen saturation in comatose patients resuscitated from a cardiac arrest. Resuscitation.

[29] Souter M.J., Andrews P.J., Alston R.P. (1998). Jugular venous desaturation following cardiac surgery. Br. J. Anaesth..

[30] Andrews P., Dearden N.M., Miller J.D. (1991). Jugular bulb cannulation: Description of a cannulation technique and validation of a new continuous monitor. BJA Br. J. Anaesth..

[31] Nortje J., Gupta A.K. (2006). The role of tissue oxygen monitoring in patients with acute brain injury. Br. J. Anaesth..

[32] Lang E.W., Mulvey J.M., Mudaliar Y., Dorsch N. (2007). Direct cerebral oxygenation monitoring—A systematic review of recent publications. Neurosurg. Rev..

[33] Nemani V.M., Manley G.T. (2004). Brain tissue oxygen monitoring: Physiologic principles and clinical application. Oper. Tech. Neurosurg..

[34] Kautsky H., Merkel H. (1939). Phosphoreszenz, Selbstauslöschung und Sensibillisatorwirkung organischer Stoffe. Naturwissenschaften.

[35] Smith M.M.T. (2007). Multimodal monitoring in traumatic brain injury: Current status and future directions. Br. J. Anaesth..

[36] Schmidt J.M., Claassen J. (2012). Clinical utility of brain tissue oxygen tension in treatment of brain injury more complicated than it appears. Clin. Neurophysiol..

[37] Hong X.Q., Feng H. (2004). Monitoring of brain tissue oxygen in treatment of brain injury. J. Trauma. Surg..

[38] Jaeger M., Soehle M., Meixensberger J. (2005). Brain tissue oxygen (PtiO2): A clinical comparison of two monitoring devices. Acta Neurochirurgica. Suppl..

[39] Yang Y., Chang T., Luo T., Li L., Qu Y., Neurosurgery D.O., Hosipital T. (2017). Multimodal Monitoring for Severe Traumatic Brain Injury. Med. Recapitul..

[40] Forcione M., Ganau M., Prisco L., Chiarelli A.M., Bellelli A., Belli A., Davies D.J. (2021). Mismatch between Tissue Partial Oxygen Pressure and Near-Infrared Spectroscopy Neuromonitoring of Tissue Respiration in Acute Brain Trauma: The Rationale for Implementing a Multimodal Monitoring Strategy. Int. J. Mol. Sci..

[41] Saldien V., Schepens T., Vanlinthout L., Wildemeersch D., Wouters K., Vercauteren M., Menovsky T. (2020). Real-time Monitoring of Cerebral Blood Flow and Cerebral Oxygenation During Rapid Ventricular Pacing in Neurovascular Surgery: A Pilot Study. J. Neurosurg. Anesthesiol..

[42] Erecińska M., Silver I.A. (2001). Tissue oxygen tension and brain sensitivity to hypoxia. Respir. Physiol..

[43] Zauner A., Doppenberg E.M.R., Woodward J.J., Choi S.C., Young H.F., Bullock R. (1997). Continuous Monitoring of Cerebral Substrate Delivery and Clearance: Initial Experience in 24 Patients with Severe Acute Brain Injuries. Neurosurgery.

[44] Kiening K.L., Unterberg A.W., Bardt T.F., Schneider G.-H., Lanksch W.R. (1996). Monitoring of cerebral oxygenation in patients with severe head injuries: Brain tissue PO2 versus jugular vein oxygen saturation. J. Neurosurg..

[45] Sun H., Zheng M., Wang Y., Diao Y., Zhao W., Wei Z. (2016). Brain tissue partial pressure of oxygen predicts the outcome of severe traumatic brain injury under mild hypothermia treatment. Neuropsychiatr Dis. Treat..

[46] Doppenberg E., Zauner A., Bullock R., Ward J.D., Young H.F. (1998). Correlations between brain tissue oxygen tension, carbon dioxide tension, pH, and cerebral blood flow—a better way of monitoring the severely injured brain?. Surg. Neurol..

[47] Xie Q., Wu H.-B., Yan Y.-F., Liu M., Wang E.-S. (2017). Mortality and Outcome Comparison Between Brain Tissue Oxygen Combined with Intracranial Pressure/Cerebral Perfusion Pressure–Guided Therapy and Intracranial Pressure/Cerebral Perfusion Pressure–Guided Therapy in Traumatic Brain Injury: A Meta-Analysis. World Neurosurg..

[48] Bouzat P., Sala N., Payen J.F., Oddo M. (2013). Beyond intracranial pressure: Optimization of cerebral blood flow, oxygen, and substrate delivery after traumatic brain injury. Ann. Intensive Care.

[49] Figaji A.A., Zwane E., Fieggen A.G., Argent A.C., Roux P., Peter J.C. (2010). The Effect of Increased Inspired Fraction of Oxygen on Brain Tissue Oxygen Tension in Children with Severe Traumatic Brain Injury. Neurocritical Care.

[50] Nemer S.N., Caldeira J.B., Santos R.G., Guimarães B.L., Garcia J.M., Prado D., Silva R.T., Azeredo L.M., Faria E.R., Souza P.C.P. (2015). Effects of positive end-expiratory pressure on brain tissue oxygen pressure of severe traumatic brain injury patients with acute respiratory distress syndrome: A pilot study. J. Crit. Care.

[51] Lyson T., Sieskiewicz A., Rutkowski R., Rybaczek M., Mariak Z. (2020). Brain tissue oxygenation during transnasal endoscopic skull base procedures. Adv. Med. Sci..

[52] Domínguez-Roldán J.M., Lubillo S., Videtta W., Llompart-Pou J.A., Badenes R., Márquez Rivas J., Ibáñez J., Godoy D.A., Murillo-Cabezas F., Lagares Gómez-Abascal A. (2020). International consensus on the monitoring of cerebral oxygen tissue pressure in neurocritical patients. Neurocirugía.

[53] Dings J., Meixensberger J., Jäger A., Roosen K. (1998). Clinical Experience with 118 Brain Tissue Oxygen Partial Pressure Catheter Probes. Neurosurgery.

[54] Ghosh A., Elwell C., Smith M. (2012). Cerebral Near-Infrared Spectroscopy in Adults: A Work in Progress. Anesth. Analg..

[55] Shaaban-Ali M., Momeni M., Denault A. (2021). Clinical and Technical Limitations of Cerebral and Somatic Near-Infrared Spectroscopy as an Oxygenation Monitor. J. Cardiothorac. Vasc. Anesth..

[56] Tak S., Ye J.C. (2014). Statistical analysis of fNIRS data: A comprehensive review. NeuroImage.

[57] Cope M. (1991). The Development of a Near Infrared Spectroscopy System and its Application for Non Invaswe Monitoring of Cerebral Blood and Tissue Oxygenation in the Newborn Infants.

[58] Cope M., Delpy D.T. (1988). System for long-term measurement of cerebral blood and tissue oxygenation on newborn infants by near infra-red transillumination. Med. Biol. Eng. Comput..

[59] Cour A.L., Greisen G., Hyttel-Sørensen S. (2018). In Vivo validation of cerebral near-infrared spectroscopy: A review. Neurophotonics.

[60] Hornberger C., Wabnitz H. (2018). Approaches for calibration and validation of near-infrared optical methods for oxygenation monitoring. Biomed. Tech. Eng..

[61] Smith M. (2011). Shedding light on the adult brain: A review of the clinical applications of near-infrared spectroscopy. Philos. Trans. R. Soc. A Math. Phys. Eng. Sci..

[62] Heringlake M., Garbers C., Kbler J.H., Anderson I., Heinze H., Schn J., Berger K.U., Dibbelt L., Sievers H.H., Hanke T. (2011). Preoperative cerebral oxygen saturation and clinical outcomes in cardiac surgery. Anesthesiology.

[63] Teng Y., Ding H., Gong Q., Jia Z., Huang L. (2006). Monitoring cerebral oxygen saturation during cardiopulmonary bypass using near-infrared spectroscopy: The relationships with body temperature and perfusion rate. J. Biomed. Opt..

[64] Parnia S., Yang J., Nguyen R., Ahn A., Zhu J., Inigo-Santiago L., Nasir A., Golder K., Ravishankar S., Bartlett P. (2016). Cerebral Oximetry During Cardiac Arrest: A Multicenter Study of Neurologic Outcomes and Survival. Crit. Care Med..

[65] Wally D., Velik-Salchner C. (2015). Near-infrared spectroscopy during cardiopulmonary resuscitation and mechanical circulatory support. Med. Klin. Intensivmed. Notf..

[66] Dunham C.M., Ransom K.J., Flowers L.L., Siegal J.D., Kohli C.M. (2004). Cerebral hypoxia in severely brain-injured patients is associated with admission Glasgow Coma Scale score, computed tomographic severity, cerebral perfusion pressure, and survival. J. Trauma.

[67] Yokose N., Sakatani K., Murata Y., Awano T., Igarashi T., Nakamura S., Hoshino T., Katayama Y. (2010). Bedside Monitoring of Cerebral Blood Oxygenation and Hemodynamics after Aneurysmal Subarachnoid Hemorrhage by Quantitative Time-Resolved Near-Infrared Spectroscopy. World Neurosurg..

[68] Samra S.K., Dy E.A., Welch K., Dorje P., Zelenock G.B., Stanley J.C. (2000). Evaluation of a Cerebral Oximeter as a Monitor of Cerebral Ischemia during Carotid Endarterectomy. Anesthesiology.

[69] Moritz S., Kasprzak P., Arlt M., Taeger K., Metz C. (2007). Accuracy of Cerebral Monitoring in Detecting Cerebral Ischemia during Carotid Endarterectomy. Anesthesiology.

[70] Alrawi P.G., Kirkpatrick P.J. (2006). Tissue Oxygen Index: Thresholds for cerebral ischemia using near-infrared spectroscopy. Stroke.

[71] Srensen H., Grocott H.P., Secher N.H. (2016). Near infrared spectroscopy for frontal lobe oxygenation during non-vascular abdominal surgery. Clin. Physiol. Funct. Imaging.

[72] Nielsen H.B. (2014). Systematic review of near-infrared spectroscopy determined cerebral oxygenation during non-cardiac surgery. Front. Physiol..

[73] Trafidło T., Gaszyński T., Gaszyński W., Nowakowska-Domagała K. (2015). Intraoperative monitoring of cerebral NIRS oximetry leads to better postoperative cognitive performance: A pilot study. Int. J. Surg..

[74] Viola S., Viola P., Buongarzone M.P., Fiorelli L., Litterio P. (2013). Tissue oxygen saturation and pulsatility index as markers for amnestic mild cognitive impairment: NIRS and TCD study. Clin. Neurophysiol..

[75] Katheria A.C., Stout J., Morales A.L., Poeltler D., Rich W.D., Steen J., Nuzzo S., Finer N. (2021). Association between early cerebral oxygenation and neurodevelopmental impairment or death in premature infants. J. Perinatol..

[76] Tran N.N., Tran M., Elgabalawy E., Lopez J., Kysh L. (2020). The use of near-infrared spectroscopy (NIRS) to measure cerebral oxygen saturation during body position changes on infants less than one year old. J. Pediatric Nurs..

[77] Chock V.Y., Kwon S.H., Ambalavanan N., Batton B., Meurs K. (2020). Cerebral Oxygenation and Autoregulation in Preterm Infants (Early NIRS Study). J. Pediatrics.

[78] Ozawa J., Watanabe T., Ito M., Miyake F., Nagano N., Ogawa R., Matsumura S., Araki R., Tamura M., Namba F. (2020). Defining the reference range of regional cerebral tissue oxygen saturation using a new portable near-infrared spectroscopy device for term infants. Early Hum. Dev..

[79] Berens R.J., Stuth E., Robertson F.A., Jaquiss R.D., Litwin S.B. (2010). Near infrared spectroscopy monitoring during pediatric aortic coarctation repair. Paediatr. Anaesth..

[80] Costa F.G., Hakimi N., Van Bel F. (2021). Neuroprotection of the Perinatal Brain by Early Information of Cerebral Oxygenation and Perfusion Patterns. Int. J. Mol. Sci..

[81] Siegemund M., Bommel J.V., Ince C. (1999). Assessment of regional tissue oxygenation. Intensive Care Med..

[82] Barud M., Dabrowski W., Siwicka-Gieroba D., Robba C., Bielacz M., Badenes R. (2021). Usefulness of Cerebral Oximetry in TBI by NIRS. J. Clin. Med..

[83] Strangman G., Boas D.A., Sutton J.P. (2002). Non-invasive neuroimaging using near-infrared light. Biol. Psychiatry.

[84] Kobayashi K., Kitamura T., Kohira S., Torii S., Mishima T., Ohkubo H., Tanaka Y., Sasahara A., Fukunishi T., Ohtomo Y. (2018). Cerebral oximetry for cardiac surgery: A preoperative comparison of device characteristics and pitfalls in interpretation. J. Artif. Organs.

[85] McCredie V.A., Chavarría J., Baker A.J. (2021). How do we identify the crashing traumatic brain injury patient—the intensivist’s view. Curr. Opin. Crit. Care.

[86] Wintermark M., Sesay M., Barbier E., Borbély K., Yonas H. (2005). Comparative Overview of Brain Perfusion Imaging Techniques. Stroke.

[87] Magistretti P., Pellerin L., Rothman D.L. (1999). Energy on Demand. Science.

[88] René K., Andreas R., Elke H., Andrea S., Hilal Y., Elvis H., Michael Z., Volker S. (2004). Functional Magnetic Resonance Imaging-integrated Neuronavigation: Correlation between Lesion-to-Motor Cortex Distance and Outcome. Neurosurgery.

[89] Soschinski J., Mine L.B., Geraskin D., Bennink G., Kohlbareis M. (2007). Cerebral oxygenation monitoring during cardiac bypass surgery in infants with broad band spatially resolved spectroscopy. Advances in Medical Engineering.

[90] Guerrero-Mosquera C., Borragán G., Peigneux P. (2016). Automatic detection of noisy channels in fNIRS signal based on correlation analysis. J. Neurosci. Methods.

[91] Kamran M.A., Hong K.-S. (2014). Reduction of physiological effects in fNIRS waveforms for efficient brain-state decoding. Neurosci. Lett..

[92] Denault A., Deschamps A., Murkin J.M. (2007). A Proposed Algorithm for the Intraoperative Use of Cerebral Near-Infrared Spectroscopy. Semin. Cardiothorac. Vasc. Anesth..

[93] Jenny C., Biallas M., Trajkovic I., Fauchere J.C., Bucher H.U., Wolf M. (2011). Reproducibility of cerebral tissue oxygen saturation measurements by near-infrared spectroscopy in newborn infants. J. Biomed. Opt..

[94] Zonios G., Bykowski J., Kollias N. (2001). Skin melanin, hemoglobin, and light scattering properties can be quantitatively assessed in vivo using diffuse reflectance spectroscopy. J. Investig. Dermatol..

[95] Matas A., Sowa M.G., Taylor G., Mantsch H.H. (2002). Melanin as a confounding factor in near infrared spectroscopy of skin. Vib. Spectrosc..

[96] Boulnois J.L. (1986). Photophysical processes in recent medical laser developments: A review. Lasers Med. Sci..

